# *Tobacco Mosaic Virus* in the Lungs of Mice following Intra-Tracheal Inoculation

**DOI:** 10.1371/journal.pone.0054993

**Published:** 2013-01-30

**Authors:** Fanny Balique, Philippe Colson, Abdoulaye Oury Barry, Claude Nappez, Audrey Ferretti, Khatoun Al Moussawi, Tatsiana Ngounga, Hubert Lepidi, Eric Ghigo, Jean-Louis Mege, Hervé Lecoq, Didier Raoult

**Affiliations:** 1 Aix-Marseille Univ., Unité de Recherche sur les Maladies Infectieuses et Tropicales Émergentes (URMITE) UM 63 CNRS 7278 IRD 3R198 INSERM U1095, IHU Méditerranée Infection, Facultés de Médecine et de Pharmacie, Marseille, France; 2 Pôle des Maladies Infectieuses et Tropicales Clinique et Biologique, Fédération de Bactériologie-Hygiène-Virologie, Centre Hospitalo-Universitaire Timone, Assistance Publique des Hôpitaux de Marseille, Marseille, France; 3 Institut National de la Recherche Agronomique (INRA), UR 407, Pathologie Végétale, Montfavet, France; University of Utah School of Medicine, United States of America

## Abstract

Plant viruses are generally considered incapable of infecting vertebrates. Accordingly, they are not considered harmful for humans. However, a few studies questioned the certainty of this paradigm. *Tobacco mosaic virus* (TMV) RNA has been detected in human samples and TMV RNA translation has been described in animal cells. We sought to determine if TMV is detectable, persists, and remains viable in the lung tissues of mice following intratracheal inoculation, and we attempted to inoculate mouse macrophages with TMV. In the animal model, mice were intratracheally inoculated with 10^11^ viral particles and were sacrificed at different time points. The virus was detected in the mouse lungs using immunohistochemistry, electron microscopy, real-time RT-PCR and sequencing, and its viability was studied with an infectivity assay on plants. In the cellular model, the culture medium of murine bone marrow derived macrophages (BMDM) was inoculated with different concentrations of TMV, and the virus was detected with real-time RT-PCR and immunofluorescence. In addition, anti-TMV antibodies were detected in mouse sera with ELISA. We showed that infectious TMV could enter and persist in mouse lungs via the intratracheal route. Over 14 days, the TMV RNA level decreased by 5 log_10_ copies/ml in the mouse lungs and by 3.5 log_10_ in macrophages recovered from bronchoalveolar lavage. TMV was localized to lung tissue, and its infectivity was observed on plants until 3 days after inoculation. In addition, anti-TMV antibody seroconversions were observed in the sera from mice 7 days after inoculation. In the cellular model, we observed that TMV persisted over 15 days after inoculation and it was visualized in the cytoplasm of the BMDM. This work shows that a plant virus, *Tobacco mosaic virus*, could persist and enter in cells in mammals, which raises questions about the potential interactions between TMV and human hosts.

## Introduction

Plant viruses are abundant inhabitants of the biosphere and are responsible for major diseases in a wide range of crops worldwide [1]. Their host repertoire is not known to overlap with that of vertebrate viruses, which suggests that the viruses of *Plantae* and *Vertebrata* exist as infectious members of two separate worlds. Accordingly, plant viruses are not considered harmful for humans. An example of the confidence in this dogma comes from new prospects in the field of vaccine immunization that use plant virus-based vaccines [2], [3].

Tobamoviruses are known for their extraordinary resistance to heat, desiccation, freezing and thawing [4]. The archetypal *Tobacco mosaic virus* (TMV) is considered to be extraordinarily stable and is the most heat-resistant plant pathogen known [5], [6]. TMV remained identifiable by electron microscopy after a storage of 50 years [7]. TMV has a single-stranded RNA genome of 6,400 nucleotides and was recently classified in the *Virgaviridae* family [8]. This rod-shaped virus infects tobacco plants and causes mottling and discoloration of leaves. The abundance of biological data accumulated for TMV [9], its high replication rate in plants, and the dogma that TMV, as other plant viruses, is safe for vertebrate animals including humans, led researchers to consider this virus as a good candidate for new experimental vaccine strategies [2], [3], [10]–[13]. Indeed, TMV-derived recombinant vaccines can facilitate the exposure of vertebrates to various peptides.

However, TMV RNA entry and translation have been described in oocytes of *Xenopus laevis*, in mouse liver mitochondria and possibly in rabbit reticulocytes [14]–[16]. In addition, TMV capsid protein has been translated in HeLa cells in which TMV RNA was demonstrated to trigger autophagy, and intracellular TMV-resembling particles were reported by electron microscopy [17]. Moreover, several teams reported the presence of TMV in non-human and human vertebrates. Erickson et al. described in 1953 the persistence of TMV in extracts of mouse livers 9 days after intravenous inoculation with a 2 mg dose of TMV by means of precipitation with anti-TMV antibodies, and 15 days post-injection by electron microscopy [18]. The same team demonstrated in 1957 the intracellular localization of TMV in the mouse liver [19]. Later, in the 1960s, Bothwell raised the issue of a possible causal relationship between TMV and lung cancer [5], and TMV has been cultured from the sputum and thoracentesis fluids of cigarette smokers with a history of pulmonary disease, including lung cancer [20], [21]. Finally, very recently, TMV RNA was recovered by two different teams using metagenomics in the feces of non-diarrheic and diarrheic persons [22], [23]. The clinical significance of these findings is unresolved. Detecting TMV in human feces is reasonable as we may ingest TMV particles through eating TMV-infected vegetables, e.g., peppers or tomatoes. Tobacco cigarette smoking may be another major source of exposure to TMV. Indeed, according to the world health organization, more than 15 billion cigarettes are smoked worldwide daily (http://www.who.int/tobacco/en/atlas8.pdf) and TMV is known to be present and stable in the tobacco of cigarettes and cigars [5], [6], [24], [25]. We recently detected TMV RNA in all 47 smoking cigarettes of six brands, mean titer being 9.5 log_10_ copies/cigarette [25]. In addition, TMV was found viable in 53% of these cigarettes as shown by the development of local lesions on leaves of Nicotiana tabacum Xanthi (NtX) after inoculation. Moreover, we detected TMV RNA in 45% of the 44 saliva from 12 smokers while in none of the 16 saliva from 15 non-smokers (p = 0.001), which indicated that the TMV genome may get access to the human body through smoking. Interestingly, Bousbia et al. recently reported the protracted detection of plant DNA, including *Nicotiana tabacum* chloroplast DNA, in the bronchoalveolar lavage fluid of mechanically ventilated pneumonia patients, which suggests that TMV may be conveyed to the lungs in tobacco [26]. To better understand the interactions between TMV and humans, we sought to determine if TMV is detectable, persists, and remains viable in the lung tissues of vertebrate animals following inoculation. For this purpose, we used an experimental mouse model consisting of intratracheal inoculation of the virus. In addition, we attempted to infect mouse macrophages with TMV.

## Results

### TMV Localization in Mouse Lungs

**Figure 1:**
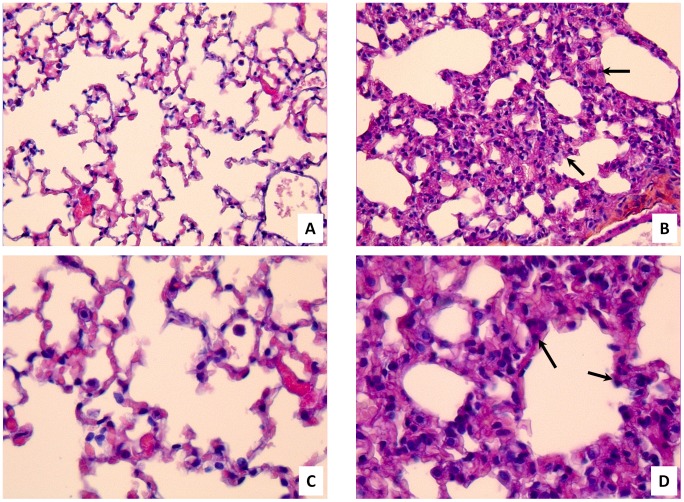
Lung sections from water-inoculated mouse (A and C), and TMV-inoculated mouse at day 3 (B and D) after intratracheal inoculation. Note the absence of inflammation in the lungs of control mice, whereas inflammatory infiltrates in interalveolar walls composed in part by macrophages were observed in lungs from TMV-inoculated mice. Hematoxylin-eosin staining was used. Magnification, 200X (A and B) and 400X (C and D). Arrows indicate the inter-alveolar walls inflammation.

**Figure 2:**
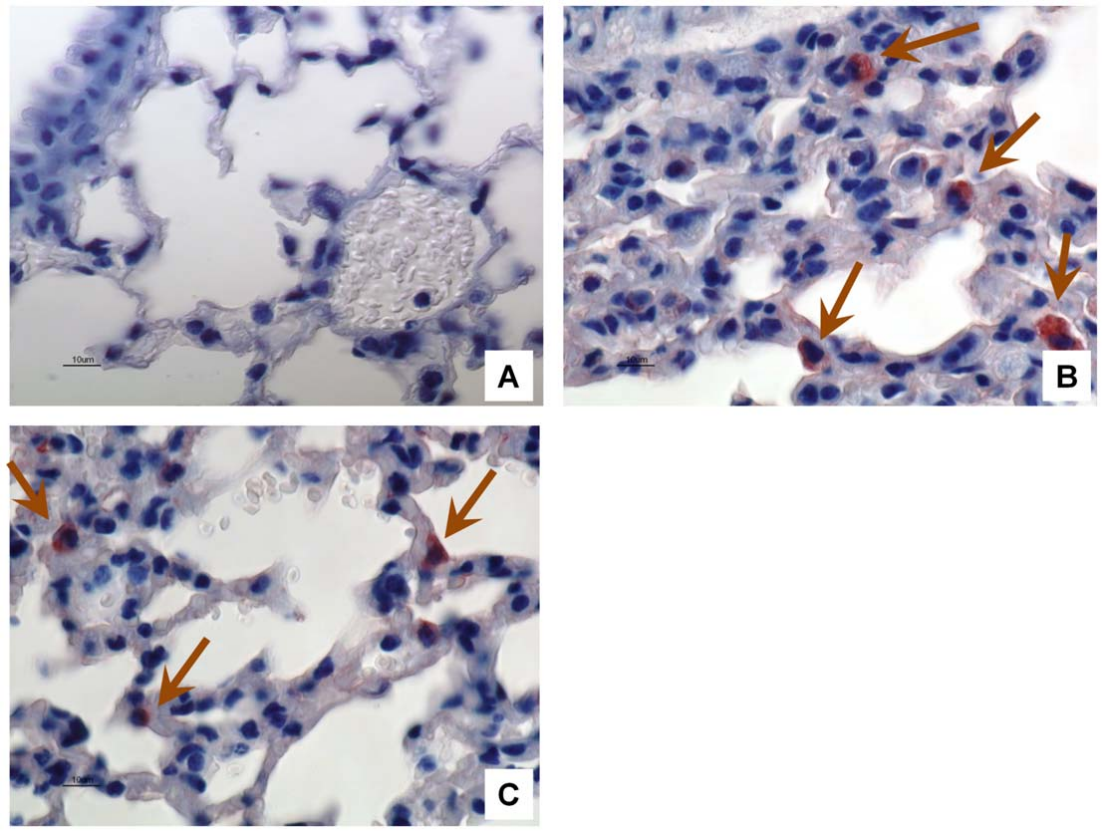
Detection of TMV antigen by immunohistochemistry in lungs of TMV-inoculated mice. No immunodetection was observed in lung from a water-inoculated mouse (A), whereas cytoplasmically immunopositive cells located in inflammatory infiltrates present in interalveolar walls, most likely macrophages, were detected at day 1 (B) and day 3 (C) after intratracheal inoculation. Polyclonal rabbit anti-TMV antibody was used at a dilution of 1∶500, with hemaxylin counterstain. Magnification, 400X. Arrows show macrophages positive for TMV antigen detection.

At different times after intratracheal inoculation, TMV-inoculated and control mice were sacrificed and their lungs were collected. Inflammatory reactions to TMV were observed in lungs of all three inoculated mice at day 3 after intra-tracheal inoculation, whereas no histological changes were found in the two control mice at different times and in other TMV-inoculated mice at day 1, 7 and 14 after the virus inoculation (Figure 1). In TMV-inoculated mice at day 3, inflammatory infiltrates without necrotic damage were confined within the alveolar walls. The interalveolar walls were infiltrated by mononuclear inflammatory cells composed mainly of macrophages without granulomatous organization. The bronchoalveolar air spaces were relatively free of cellular exudates. TMV antigens were detected by immunohistochemistry in the lungs of one TMV-inoculated mouse at day 1 and in two mice at day 3 after inoculation whereas not in control mice and in TMV-inoculated mice at days 7 and 14 post-inoculation (Figure 2). Immunopositive material was observed in the cytoplasm of cells that had macrophage morphology.

### Anti-TMV Serologies

**Figure 3:**
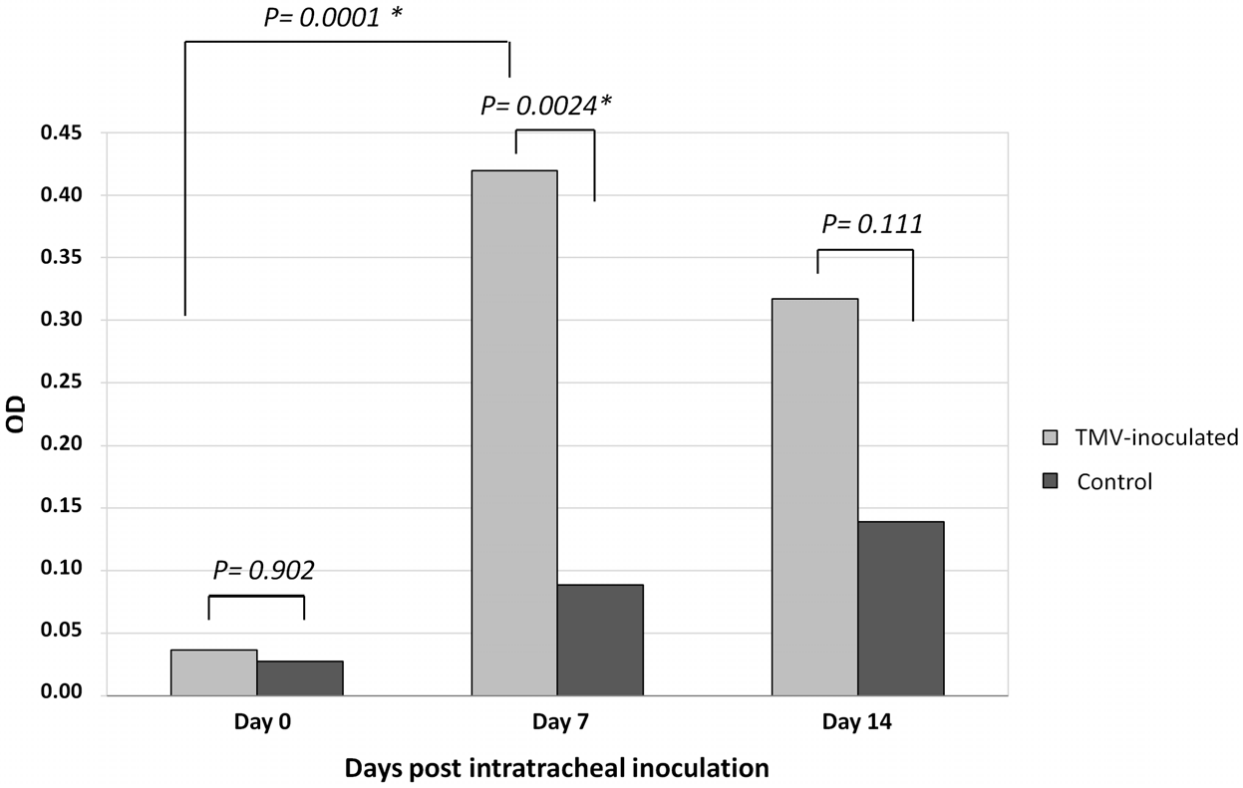
Anti-TMV antibody testing in mouse serum samples. Detection of anti-TMV total antibodies in serum samples of 10 TMV-inoculated and 5 control mice at day 0 (just before the operation), and 13 TMV-inoculated and 8 control mice at day 7 and day 14 after intratracheal inoculation. *Statistically significant.

Anti-TMV total antibodies were concurrently tested in serum samples from TMV-inoculated mice and control mice immediately before the intratracheal inoculation and 7 and 14 days after inoculation (Figure 3). For TMV-inoculated mice, the mean optical density (OD) was 0.04±0.03 at day 0, 0.42±0.23 at day 7 and 0.32±0.21 at day 14 post-inoculation. The mean OD increase between day 0 and day 7 was statistically significant (p = 0.0001). In contrast, for serum samples from control mice, the mean OD was 0.03±0.01 at day 0, 0.09±0.05 at day 7 and 0.14±0.04 at day 14 post-inoculation. Furthermore, we found a statistically significant difference between mean OD for sera collected from TMV-inoculated and control mice 7 days after TMV inoculation (p = 0.0024). Finally, taking into account longitudinal serum samples from ten different mice in the same experiment, seroconversion was observed in 6 TMV-inoculated mice on day 7, based on an OD greater than the mean value at baseline plus 10 SD [27], [28].

### Detection, Quantification and Viability of TMV in Mice Samples

#### Mouse lungs

**Figure 4:**
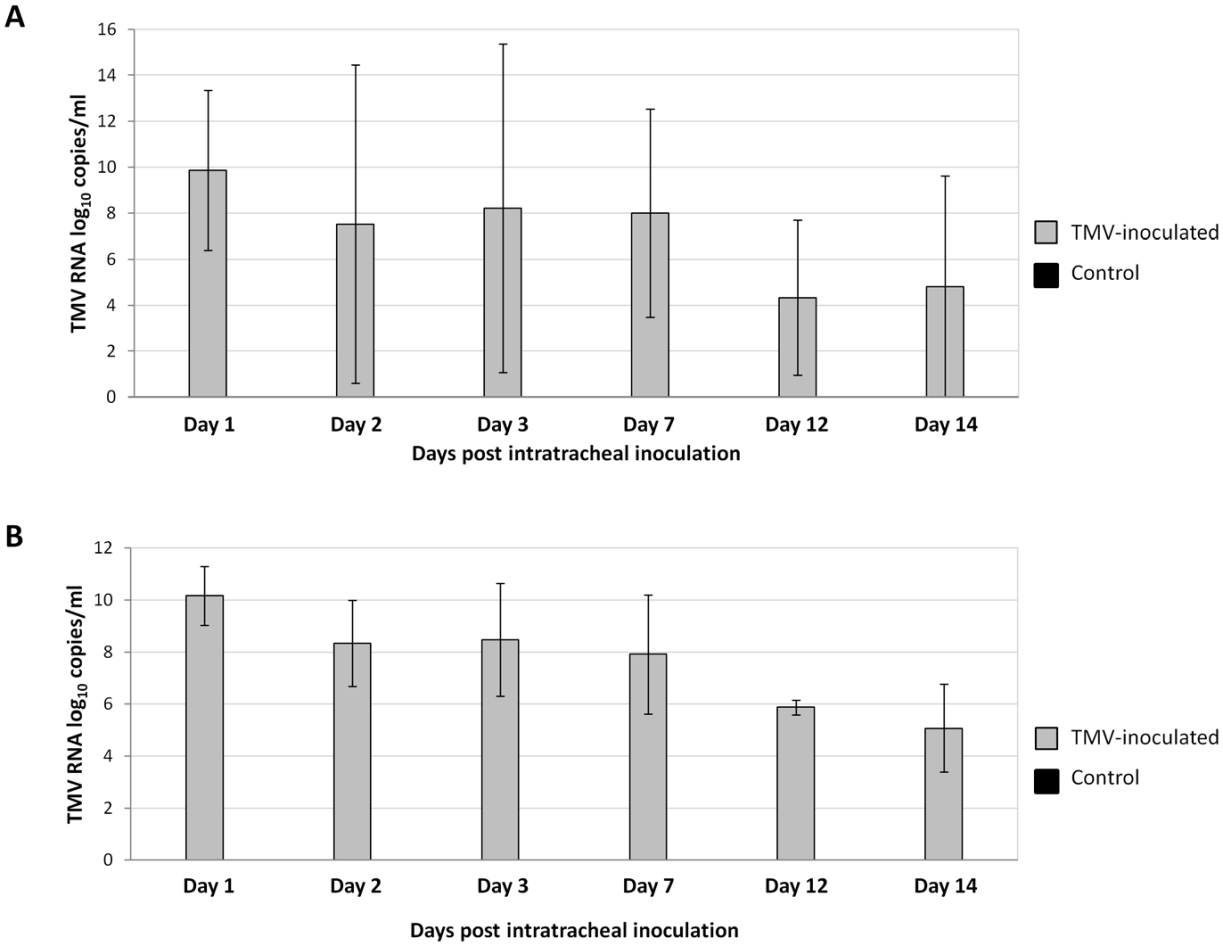
TMV RNA quantification in mouse lungs by real-time RT-PCR. A: TMV coat protein gene system; B: TMV replicase gene system. Control, non-inoculated mice.

**Figure 5:**
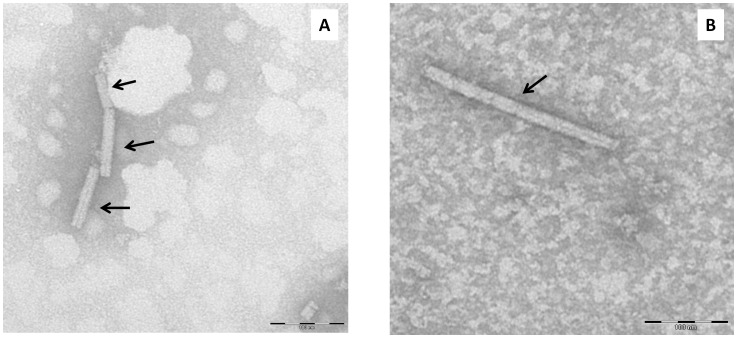
TMV-like particles observed by negative coloration on electron microscopy in the homogenate of one inoculated lung sample 1 day (A) and 3 days (B) post-inoculation. Arrows indicate TMV-like particles.

After mouse sacrifice, a sample of lung tissue was crushed for RNA extraction, and TMV RNA was quantified by real-time RT-PCR targeting the coat protein and replicase genes (Table 1). TMV RNA was detected by two real-time RT-PCR assays in the lungs of all 21 TMV-inoculated mice from day 1 until 14 days after TMV inoculation. In contrast, no TMV RNA was detected in the 11 samples from control mice. The mean TMV RNA level assessed in lungs from TMV-inoculated mice with the system targeting the coat protein was 9.87±3.48 log_10_ copies/ml one day after TMV inoculation, 7.5±6.9 log_10_ at day 2, 8.2±7.2 log_10_ at day 3, 8.0±4.5 log_10_ at day 7, 4.3±3.4 log_10_ at day 12 and 4.8±4.8 log_10_ at day 14 (Figure 4). Similar results were obtained using the real-time PCR system for the replicase gene. Indeed, the TMV RNA level was 10.2±1.1 log_10_ copies/ml one day after TMV inoculation, 8.3±1.7 log_10_ at day2, 8.5±2.2 log_10_ at day3, 7.9±2.3 log_10_ at day 7, 5.9±0.3 log_10_ at day12 and 5.1±1.7 log_10_ at day14 (Figure 4). Thus, in 14 days, the TMV RNA level decreased by approximately 5 log_10_ in mouse lung homogenates. In addition, all lung samples were tested by conventional RT-PCR for the helicase gene or the protein movement gene (Table 1). In 20 of 21 lung samples of TMV-inoculated mice, TMV RNA was identified by population sequencing. Additionally, rod shape TMV-resembling particles could be visualized by electron microscopy in 7 lung homogenate samples of 17 TMV-inoculated mice; two lung samples were observed positives at day 1, one at day 2, two at day 3 and two at day 7 after TMV inoculation (Figure 5).

**Table 1:** Probes and primers used for RT-PCR assays.

RT-PCR type	Probe/primer	Name	Sequence	Position (nt)^a^	Gene
Real-time	Probe	TMV_Mars_Rep1	AAA CCC TTG CAT GGC AAG ATC CTG A	3,099–3,123	Replicase
	Forward primer	TMV_Mars_RepFwd1	CCG TGA TCA ATC CGA TCT CA	3,079–3,098	
	Reverse primer	TMV_Mars_RepRev1	TGT CTC GCC TTG CAC TTC AT	3,206–3,187	
Real-time	Probe	TMV_Mars_CP1	CAG TGA GGT GTG GAA ACC TTC ACC ACA	5,858–5,881	Coat protein
	Forward primer	TMV_Mars_CPFwd1	CAA GCT GGA ACT GTC GTT CA	5,829–5,848	
	Reverse primer	TMV_Mars_CPRev1	CGG GTC TAA YAC CGC ATT GT	5,948–5,929	
Conventional	Forward primer	vs-S12.12Fwd	TGG CkA AAC TCA GrA CTC TG	2,495–2,515	Helicase
	Reverse primer	vs-S12.12Rev	AAA TTm ACA CAA CCA GTA TGC A	2,831–2,853	
Conventional	Forward primer	TMV 1Fwd	CCT GAC AAA AAT GGA GAA GAT	4,950–4,970	Movement protein
	Reverse primer	TMV 2Rev	AAA GCG GAC AGA AAC CCG CT	5,368–5,349	

a in reference to sequence GenBank accession no. AB369276; nt, nucleotide.

**Figure 6:**
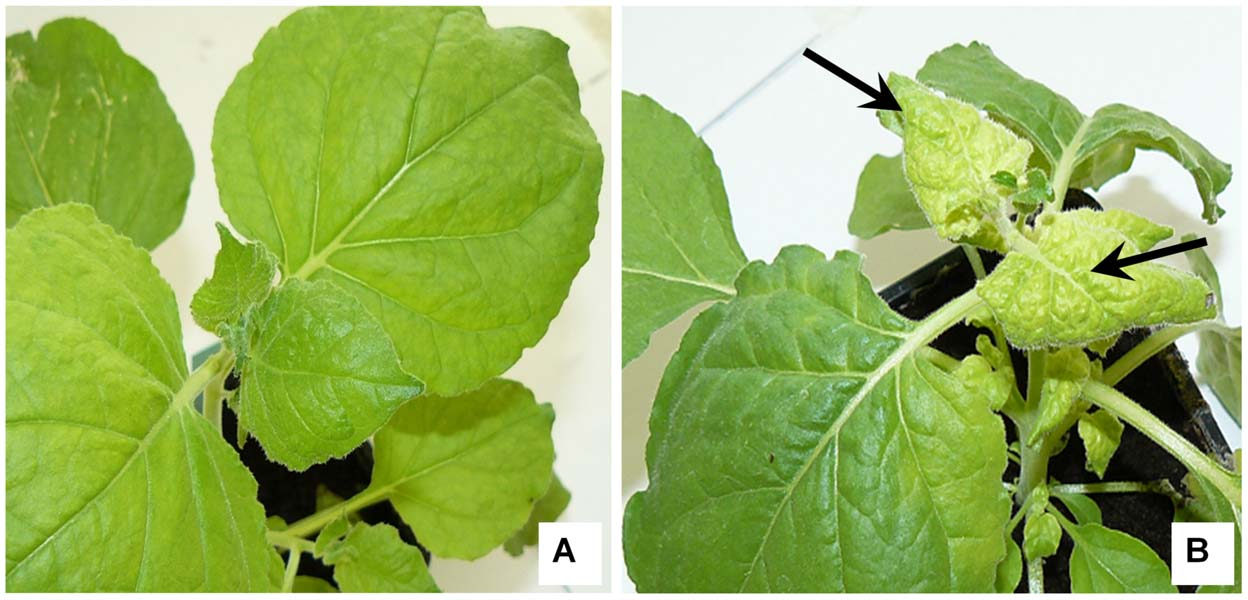
Infectivity test for TMV RNA-positive lung samples on *Nicotiana benthamiana*. A: Leaves of a non-inoculated plant; B: Leaves of a plant inoculated with a TMV RNA positive sample (TMV-inoculated mouse number 1 at day 3). Arrows show signs of systemic infection with discoloration and deformation of young leaves.

**Figure 7:**
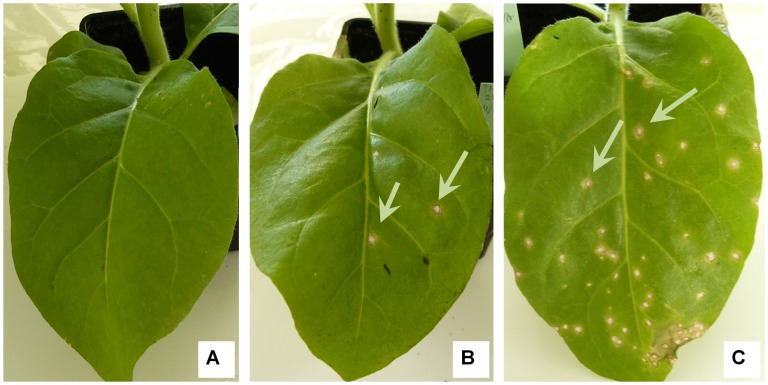
Infectivity test for TMV RNA-positive lung samples on *Nicotiana tabacum* Xanthi. A: Leaf of a non-inoculated plant; B: Leaf of a plant inoculated with a TMV RNA positive sample (TMV-inoculated mouse number 2 at day1); C: Leaf of a plant inoculated with a TMV RNA positive sample (TMV-inoculated mouse number 1 at day 3). On inoculated leaves, arrows show local necrotic lesions induced by the virus in the plant cells.

The infectivity of the TMV present in lung tissue was tested for lung samples found positive in real-time RT-PCR on hypersensitive and susceptible host plants. Necrotic local lesions were obtained on three leaves of *Nicotiana tabacum* Xanthi for three lung samples from mice inoculated with TMV including mouse no. 2 at day 1 (five local lesions), mouse no. 1 at day 3 (more than 50 local lesions) and mouse no. 3 at day 3 (one lesion). A mosaic was also obtained on *Nicotiana benthamiana* for TMV-inoculated mouse no. 1 at day 3 (Figure 6). Overall, from the 21 samples from TMV-inoculated mice tested by real-time RT-PCR, 3 (14%) induced local necrotic lesions on *Nicotiana tabacum* Xanthi, and one of them induced systemic infection in a susceptible plant host. Lung samples harvested at day 7 and day 14 were not infectious for host plants. In addition, mechanical inoculation with lung samples from control mice did not induce any signs in plants (Figure 7).

#### Bronchoalveolar fluid samples (BAL)

**Figure 8:**
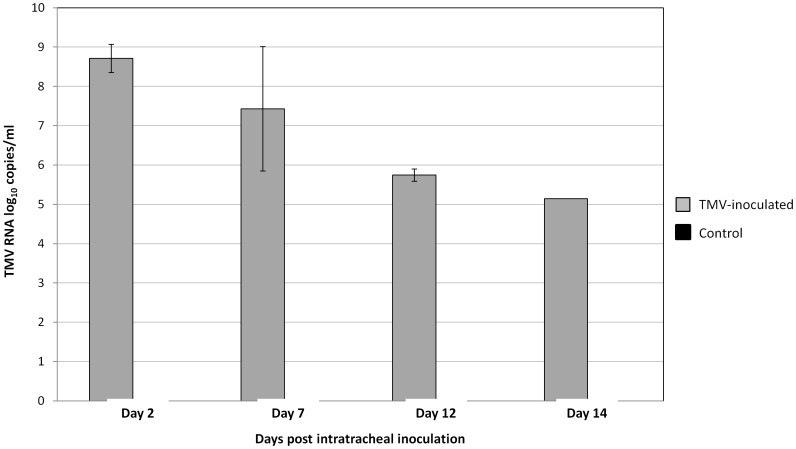
TMV RNA quantification by real-time PCR in bronchoalveolar lavage macrophages of TMV-inoculated and control mice. The PCR systems targets the coat protein gene.

BAL was performed in the second experiment on mice, immediately before the mice were sacrificed, and bronchial macrophages were isolated after two washings in RPMI with 10% fetal bovine serum (FBS). Washing supernatants, culture medium and macrophages from the BAL samples recovered from 10 TMV-inoculated mice and 5 control mice were tested by real-time RT-PCR. TMV RNA was detected in all of the samples from TMV-inoculated mice. TMV RNA titers in the BAL macrophages from TMV-inoculated mice were 8.7±0.4 log_10_ copies/ml two days post-viral inoculation, 7.4±1.6 log_10_ at day 7, 5.7±0.2 and 5.1 log_10_ at days 12 and 14 post-inoculation, respectively. Thus, the TMV RNA level decreased by 3.5 log_10_ in bronchial macrophages between days 2 and 14 post-infection (Figure 8). TMV helicase-encoding gene sequences were obtained from all BAL macrophage samples at day 2, 7, 12 and 14 after intratracheal TMV inoculation.

### Localization, Detection and Quantification of TMV in Bone Marrow Derived Macrophages (BMDM)

**Figure 9:**
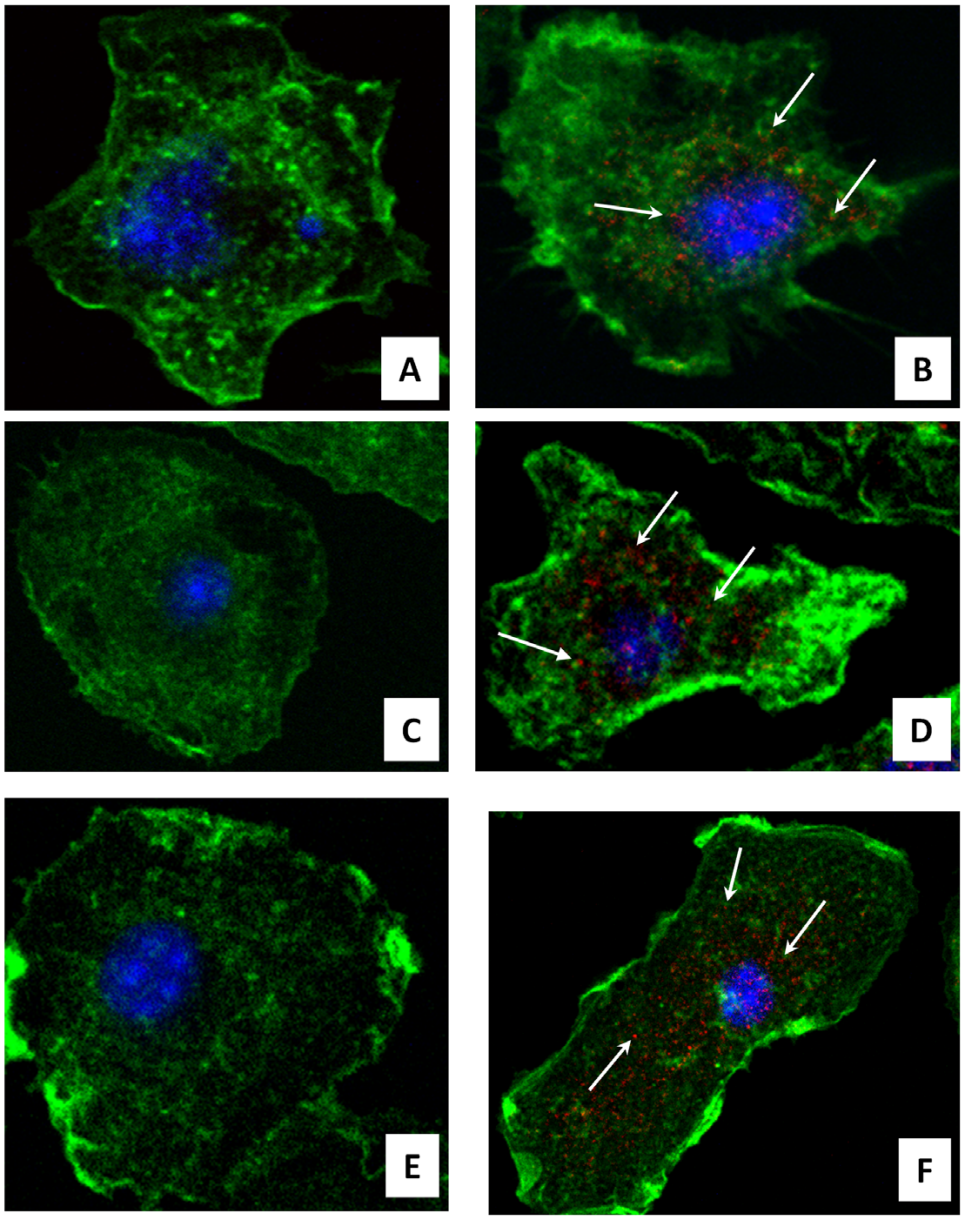
Immunofluorescence analysis of bone marrow derived macrophages (BMDM). BMDM were observed at day 1 (A and B), day 7 (C and D) and 14 (E and F) after inoculation with TMV. Non-inoculated cells (A, C, E) and TMV-inoculated cells (B, D, E) were analyzed using a mouse monoclonal anti-TMV antibody diluted at 1∶25 with goat anti-mouse 555 antibodies (red). Actin is stained with phallacidin-488 (green) and nuclei are stained with DAPI (blue). Arrows show TMV antigen (red) detected in BMDM cytoplasm. BMDM were observed by confocal microscopy with an oil immersion objective of 63X and a digital zoom of 2.

**Figure 10:**
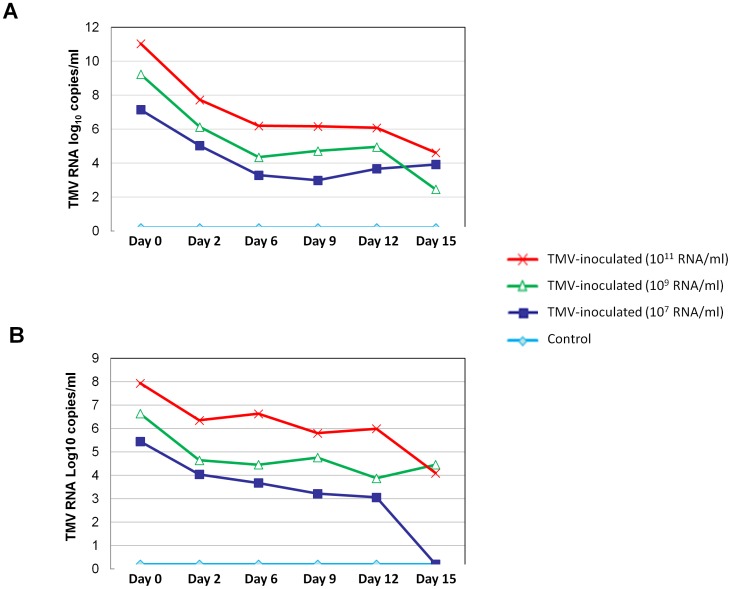
TMV RNA quantification by real-time PCR in bone marrow-derived macrophages (BMDM). Quantification of the TMV coat protein encoding RNA region in BMDM culture medium (A) and within cells (B) inoculated with 0, 10^7^,10^9^ or10^11^TMV particles during 15 days.

To further study the TMV entry into murine macrophages, we inoculated BMDM with the virus. Four hours, 1, 3, 7 and 14 days after TMV inoculation in culture medium, cells were thoroughly washed and then fixed for immunofluorescence assays. Using mouse monoclonal anti-TMV antibodies, viral antigens were localized in the cytoplasm of TMV-inoculated macrophages and were not found in non-inoculated cells (Figure 9). To assess viral persistence in murine macrophages, TMV was detected in media and in washed BMDM cells during 15 days after inoculation (Figure 10). No difference between the viability of virus-inoculated and non-inoculated cells was observed throughout the experiment at each time point as assessed by optical microscopy of the cell monolayer. In the culture media, for the three tested quantities of virus, TMV RNA titers decreased from day 0 to day 15 after inoculation. Titers decreased by 6.4 log_10_ for 10^11^ particles per well, by 6.8 log_10_ for 10^9^ particles per well, and by 3.2 log_10_ for 10^7^ particles per well. No TMV RNA was detected in non-inoculated samples. In the washed BMDM cells, TMV RNA was detected in inoculated cells, and the titer also decreased over time. Titers decreased by 3.8 log_10_ in BMDM cells for 10^11^ particles per well, by 2.2 log_10_ for 10^9^ particles per well, and at day 15 post-inoculation, TMV RNA was not detected in BMDM cells for 10^7^ particles per well. The non-inoculated BMDM control cells all tested negative for the presence of TMV RNA.

## Discussion

In this study, we showed that TMV could enter and persist in the lungs of intratracheally inoculated mice. We found TMV RNA at high titers using real-time RT-PCR in lung tissues and bronchoalveolar fluids, with RNA loads greater than 7 log_10_ copies/ml on days 2 and 7 post-inoculation, and we showed viral persistence for up to 14 days after inoculation, at which time the last mice were sacrificed. In addition, TMV-like particles were observed with electron microscopy, and immunohistochemical analysis indicated the presence of TMV in the mouse lungs. Notably, viruses could be localized within macrophages of the pulmonary tissue. Moreover, TMV remained infectious for its plant host up to 3 days post-inoculation. Finally, TMV-specific antibodies were detected in the sera of most inoculated mice. In addition, our cellular model using BMDM confirmed that TMV could be found inside animal cells, suggesting that TMV might be internalized by macrophages. We showed that TMV was localized in the cytoplasm of murine macrophages and persisted until 15 days after infection.

Although we did not evidence TMV replication in mouse lungs or murine macrophages in the present work, our findings raise concerns about the potential interactions between TMV and human hosts. There is other published literature that challenges the dogma of the strict boundaries between plants and vertebrates for viruses. In non-vertebrate animals, it was shown that plant pathogenic viruses displayed complex interactions with insects, and the transcription and replication of some plant viruses within insects was described [29]–[32]. In addition, in some cases, insects were found to be affected by plant viruses [33]. Furthermore, it was recently shown that *Tomato spotted wilt virus* (TSWV) could infect two human cell lines, HeLa and diploid fibroblasts, depending on the expression of a viral polymerase-bound host factor [34]. Additionally, despite plant virus replication was not observed in animals, *Cowpea mosaic virus* (CPMV), a plant comovirus in the picornavirus superfamily, was able to bind and enter mammalian cells, including endothelial cells, and the binding protein for the virus was identified as a cell-surface form of the intermediate filament vimentin [35]. Furthermore, CPMV was found to persist for several days post oral or intravenous inoculation in a wide panel of body tissues in mice, including in the lung and the liver [36]. Additionally, it was demonstrated that TSWV induced a strong immune response in its insect vector *Frankliniella occidentalis* [37] and that oral administration of *Cowpea severe mosaic virus, Alfalfa mosaic virus* and chimeric plant virus particles induced a durable and systemic immune response in mice [38], [39]. In addition, Zhang et al. identified that the vast majority of the viral sequences recovered from human feces corresponded to plant pathogenic RNA viruses [22]. There, the most abundant viral sequences isolated were from *Pepper mild mottle virus* (PMMoV), with up to 10^9^ virions per gram of dry weight fecal matter, while other tobamoviruses as *Paprika mild mottle virus*, *Tomato mosaic virus*, and *Tobacco mild green mosaic virus* were also detected. In another study, high-throughput sequencing also identified PMMoV RNA and other plant virus-derived sequences in 3 of the 4 fecal specimens collected from adult patients during norovirus outbreaks [23]. Very recently, we confirmed that a high PMMoV load, likely originating from food items, may be present in the human stools, and we found that PMMoV may be associated with a specific immune response and clinical symptoms, possibly imputable to spicy food, including fever, abdominal pains, and pruritus [40].

Until now, a major issue that precluded considering plant viruses as potential pathogens in humans was that they could not enter and replicate in vertebrate cells. A few aforementioned studies questioned the certainty of this paradigm [34], [35]. Moreover, mechanisms other than replication-induced pathogenicity may exist. For example, an alternative process may consist of deregulations through the micro-RNA (miRNA) regulatory pathway, which has been involved in pathogenicity in plants and in humans. Indeed, Bazzini et al. found that viruses that produce the most severe symptoms on tobacco (TMV and *Tomato mosaic virus*) altered miRNA accumulation to a greater extent than did viruses that produce mild symptoms (*Tobacco etch virus* and *Potato virus Y*) [41]. Additionally, the silencing suppressor activity of the TMV 126-kDa small replicase subunit is correlated with increased virus induced symptoms in tobacco [42] and it has been described that some phytoviruses RNA-silencing suppressors including the P38 protein of *Turnip crinkle virus* (TCV), the P19 protein of *Tomato bushy stunt virus* (TBSV) and the P15 protein of *Peanut clump virus* (PCV) were functional as silencing suppressors in human Hela cells [43] Furthermore, many human cancers appear to be induced by miRNA deregulation [44]. Therefore, the issue of interactions between plant viruses and the human body may be addressed by considering non-canonical mechanisms of diseases in vertebrate animals.

In summary, our findings and previous works suggest that the boundaries between *Plantae* and *Vertebrata* viruses may be less strict than currently accepted and they prompt a reevaluation of the potential pathogenicity of plant viruses for animals with modern microbiological tools. The role of these virions may be elucidated by analyzing the reprogramming of human cells inoculated by TMV.

## Materials and Methods

### TMV Production and Purification from Tobacco Plants

*Nicotiana tabacum* Samsun plants were infected with the tmj strain of TMV by mechanical inoculation. Dried infected leaf tissue kept over CaCl_2_ were then crushed in phosphate buffer 0.01 M Na_2_HPO_4_-KH_2_PO_4_ pH 7.2 (buffer no. 1) using 1 g of leaves per 4 ml of buffer, with carborundum (75 mg/ml) and activated charcoal (75 mg/ml). Three leaves per plant were inoculated mechanically with a gloved finger before being rinsed with water. Two weeks later, young leaves with signs were collected and fresh leaves were ground into phosphate buffer no. 2 (0.5 M Na_2_HPO_4_-KH_2_PO_4_ pH 7.2) using 1 g of fresh leaves per 3 ml of buffer; 1% 2-mercapto-ethanol was added just before grinding. After homogenization, 8% (v/v) n-butanol was added to the extract and was stirred for 15 min before centrifugation at 10,000 g for 30 min at 10°C. The supernatant was filtered on cheese-cloth tissue then stirred for 15 min with polyethylene glycol (PEG) 6000 4% (w/v). After a centrifugation step at 10,000 g for 15 min at 10°C, the pellet was diluted into 10 ml of buffer no. 1. This solution was centrifuged at 10,000 g for 15 min at 10°C, and the supernatant was complemented with PEG 6000 4% (w/v) and NaCl 4% (w/v), before stirring for 15 min and centrifugating at 10,000 g for 15 min at 10°C. The pellet was diluted into 1–2 ml of buffer no. 1 and then centrifuged at 10,000 g for 15 min at 10°C. Thereafter, the supernatant was ultracentrifuged for 16 h at 35,000 rpm (Beckman 70.1TI rotor) in a cesium chloride gradient (0.505 g/ml). The whitish band corresponding to the virus was collected with a needle and a syringe, then diluted into buffer no. 1 and ultracentrifuged at 38,000 rpm (Beckman 50.2Ti rotor) for 2.5 h at 10°C. Finally, the pellet containing the purified virus was resuspended into 500 µl of buffer no. 1. The viral titer was obtained by spectrometry using an extinction coefficient at 260 nm of 3 (Nanodrop 1000 Spectrophotometer).

### Mouse Inoculation with TMV

Mice were anesthetized by intra-peritoneal injection with ketamine/xylazine (10 µl per gram of mouse). Next, they were inoculated intratracheally with 50 µl of 10^10^–10^11^ purified TMV particles, or water for control mice. TMV-inoculated mice and control (water-inoculated) mice were sacrificed at days 1, 2, 3, 7, 12 and 14 post-inoculation. The experiment of mouse inoculation with TMV was realized twice. In experiment 1, three TMV-inoculated and two water-inoculated mice were sacrificed at day 1, 3, 7 and 14 after inoculation. In experiment 2, three TMV-inoculated mice were sacrificed at day 2 and 7, two TMV-inoculated mice were sacrificed at day 12 and one was sacrificed at day 14. One water-inoculated mouse was sacrificed at day 2, 7 and 14. Bronchoalveolar lavages (BAL) were obtained in experiment 2 by injection of sterile physiological salt solution in the respiratory tract of mice followed by aspiration. To obtain macrophages, BAL were centrifuged at 300 g for 10 min at 4°C, the cellular pellet was washed in 1 ml RPMI plus 10% FBS and was incubated at 37°C for 3 h in 1 ml of RPMI plus 10% FBS. Macrophages were collected from the wells in 200 µl of PBS (1X). In addition, lungs were collected from the sacrificed mice.

### Histological and Immunohistological Analysis of TMV-infected Mouse Lung Tissues

For each mouse, lungs were removed, fixed with neutral buffered formalin (4%), and embedded in paraffin. Serial sections (3 µm) of these specimens were obtained for hematoxylin-eosin and immunohistochemical investigations. Immunohistochemical analyses were performed with a polyclonal rabbit anti-TMV antibody used at a 1∶500 dilution. The immunohistological procedure, in which an immunoperoxidase LSAB K 680 kit (Dako, Trappes, France) was used, has been described elsewhere [45].

### Anti-TMV Antibodies Detection by ELISA

In a first manipulation, anti-TMV IgG/IgM antibodies were tested in serum samples from 10 TMV-inoculated mice and 5 water-inoculated mice immediately before the intratracheal inoculation and 7 and 14 days after inoculation. In a second manipulation, anti-TMV antibodies were tested in the serum of 3 TMV-inoculated and 3 control mice sacrificed 7 and 14 days after inoculation. ELISA Nunc Maxisorp plates were coated with 100 µl of purified TMV (1 µg per well) and incubated at 4°C overnight. Thereafter, they were washed with PBS Tween 0.1% and agitated for 30 min after the addition of 200 µl of PBS with 5% milk in each well, and then the plates were washed with PBS Tween 0.1%. The serum samples were diluted to 1∶100 in PBS with 3% dried milk and 0.1% Tween. Then, 100 µl of diluted samples were added to each well and were incubated for 1 h at room temperature. The plates were washed with PBS Tween 0.1%, and then 100 µl of biotinylated anti-mouse IgG/IgM specific antibody diluted to 1∶5000 in PBS-milk 3%-Tween 0.1% were added in each well. The plates were agitated for 1 h. Thereafter, the wells were washed with PBS Tween 0.1%, and 100 µl of streptavidin HRPO conjugate CALTAG (BD Biosciences Pharmingen, Le pont de Claix, France) at 1∶10000 dilution in PBS-milk 3%-Tween 0.1% were deposited in each well. Next, the plates were washed with PBS Tween 0.1% and 200 µl of ortho-phenylenediamine was added per well. Finally, 10 min later, optical density was measured for each well at 492 nm.

### Viral RNA Extraction from Lungs

Pieces of lung were crushed in 800 µl of buffer no. 1 using a small pestle directly in the 1.5 ml microfuge tube. Then, RNA was extracted from 200 µl of the homogenates using the EZ1 virus Mini kit (version 2.0) on the BioRobot EZ1 workstation (Qiagen, Courtaboeuf, France). RNA samples were eluted in 60 µl final volume and stored at −80°C until processing. The same procedure was used for viral RNA extraction from BAL macrophages.

### TMV RNA Detection by Real-time RT-PCR

From mouse lungs, TMV RNA was detected and quantified using two systems of real-time reverse transcription (RT)-PCR, one targeting a region that corresponds to a fragment of the capsid gene and the other targeting the replicase gene (Table 1), with the SuperScript III Platinum One-Step Quantitative RT-PCR System (Invitrogen Life Technologies, Carlsbad, Calif., USA) on a Mx3000P thermocycler (Stratagene, La Jolla, CA 92037 USA) under the following conditions: 50°C for 30 min for RT, 95°C for 2 min for initial denaturation and 45 cycles including 95°C for 30 sec and 60°C for 1 minute. For each 25 µl reaction, the PCR mix contained 12.5 µl of reaction buffer, 0.75 µl of each primer (10 pmol/µl), 0.5 µl of probe, 0.5 µl of SuperScript III/RT Platinum Taq mix, 5 µl H_2_O and 5 µl of RNA extract sample. From the mouse BAL macrophages, TMV RNA was detected and quantified using the capsid gene system.

### TMV RNA Population Sequencing

TMV RNA sequences corresponding to a fragment of the helicase or the movement protein encoding gene (Table 1) were obtained by RT-PCR amplification using the SuperScript One-Step RT-PCR System (Invitrogen Life Technologies). For RT-PCR amplification, ten µl of extracted viral RNA were added to 40 µl reaction solution containing 1 µl (10 pmol/µl) of each primer, 25 µl of reaction buffer, 12 µl of water and 1 µl of RT/Platinum Taq mix. The RT-PCR reactions were conducted under the following conditions: 45°C for 30 min for RT, 94°C for 2 min for initial denaturation, followed by 40 cycles including denaturation at 94°C for 30 s, annealing at 55°C for 45 s, elongation at 72°C for 2 min, then a final elongation step at 72°C for 10 min. PCR products were purified with Sephadex G-50 Superfine on MAHVN 4550 plates (Millipore, Molsheim, France) then sequenced by using the amplification PCR primers and the Big Dye Terminator cycle sequencing kit version 1.1 on the ABI Prism 3130 genetic analyzer (Applied Biosystems, Branchburg, NJ, USA).

### Test of Infectivity with TMV

Lung samples were crushed in 1 ml of buffer no. 1. Then, 200 µl of this homogenate was mixed with carborundum and mechanically inoculated on *Nicotiana tabacum* Xanthi, which is a hypersensitive host for TMV, and on *Nicotiana benthamiana*, which is a susceptible host for TMV. In the presence of infectious TMV in the tested sample, local necrotic lesions appears on *N. tabacum* Xanthi one week after the mechanical inoculation and mosaic appears on the susceptible hosts after an additional week.

### TMV Immunofluorescence in Bone Marrow-Derived Macrophages (BMDM) after TMV Inoculation

A total of 10^5^ adherent BMDM cells per well on glass slides were inoculated with 10^10^ TMV particles. Four hours, one day, three days, 7 days and 14 days after inoculation, BMDM cells were fixed in 250 µl of 3% paraformaldehyde solution for 20 min at room temperature and were then washed with PBS 1X. Thereafter, the cells were incubated for 15 min with 250 µl of ammonium chloride NH_4_Cl (0.5 M) and were washed three times with PBS 1X. Non-inoculated cells were used as controls. A drop of 40 µl of PBS 1X solution containing 10% FBS and 0.1% saponin was applied, and the glass slides, cells side up, were incubated for 20 min in a humid chamber,. Then, a 40 µl drop of mouse monoclonal anti-TMV antibody, produced as previously described [37] and diluted 1∶25 in a solution of PBS containing 5% FCS and 0.1% saponin (solution A) was applied to the slides. These slides were incubated for 20 min in the dark in the humid chamber, followed by thorough rinsing in PBS 1X with 0.1% saponin. Another drop was applied containing secondary goat anti-mouse antibody (555 nm) diluted 1∶500 and phallacidin (actin marker, 488 nm) diluted at 1∶200 in the solution A. The slides were incubated for 20 min in the dark chamber, thoroughly rinsed in PBS 1X with 0.1% saponin, and then a drop of DAPI (diluted 1∶10000 in PBS 1X) was applied for 10 min. The slides were washed in PBS 1X with 1% saponin and then in distilled water, before being glued with 5 µl of Moviol on microscopy glass slides. The slides were dried overnight before observation with confocal microscopy (Leica TCS SP5) with sections of 0.15 µm, oil immersion objective of 63X, a numerical aperture of 1.4, and a digital zoom of 2 for an image of 1024×1024 pixels.

### TMV RNA Detection in Bone Marrow-Derived Macrophages (BMDM) after Inoculation with TMV

A total of 10^5^ bone marrow-derived macrophages (BMDM) per well were incubated overnight at 37°C in Dulbecco’s Modified Eagle’s Medium (DMEM) plus 1% glutamine, 1% penicillin-streptavidin and 10% FBS. Each well was inoculated with 20 µl of solutions containing 10^7^, 10^9^, or 10^11^ TMV particles. Non-inoculated cells were used as controls. Four hours after the inoculation, culture media were discarded and the cells were washed three times with culture medium, then 1 ml of culture medium was added to cells for incubation. The culture media and BMDM cells were collected at day 0 (4 h), 3, 6, 9, 12 and 15 after TMV inoculation. Before collection, cells were washed twice with culture medium and twice with PBS 1X before being recovered in 200 µl of PBS 1X by mechanical scraping with a tip. The culture media and BMDM cells were used for TMV detection by real-time RT-PCR.

### Statistical Analysis

Statistical analysis was performed with EpiInfo version 3.51, centers for Disease Control and prevention (CDP) Atlanta (GA,USA). Means values were compared by using either ANOVA test or Krustal-Wallis test according to the homogeneity of the variance measured by the Bartlett’s test. Differences were considered statistically significant if p value <0.05.

### Ethic Statement

This study has been approved by our institutional ethics committee for animal experiment of Aix-Marseille University (Comité d’Ethique de Marseille; Comité National de Réflexion Ethique sur l’Expérimentation Animale, no. 14), according to the guidelines of the ethics committee for animal treatment. (Agreement reference number 16-21052012).
